# Exploring the association between multidimensional social isolation and heterogeneous cognitive trajectories among older adults: evidence from China

**DOI:** 10.3389/fpubh.2024.1426723

**Published:** 2024-10-03

**Authors:** Xinlong Xie, Yanxia Lyu, Fanfan Wu, Anpeng Zong, Zhiruo Zhuang, Aijun Xu

**Affiliations:** ^1^School of Health Economics and Management, Nanjing University of Chinese Medicine, Nanjing, China; ^2^Jiangsu Research Center for Major Health Risk Management and TCM Control Policy, Nanjing University of Chinese Medicine, Nanjing, China

**Keywords:** family isolation, friend isolation, subjective isolation, heterogeneous cognitive trajectories, latent class growth models, Chinese older adults

## Abstract

**Objective:**

This study aims to elucidate the heterogeneous cognitive trajectories among older adults in China through a comprehensive, nationally representative longitudinal study. Furthermore, it seeks to investigate the impact of multidimensional social isolation on heterogeneous cognitive trajectories among older adults in China.

**Methods:**

Utilizing data from three successive waves of the Chinese Longitudinal Aging Social Survey (CLASS) spanning 2016 to 2020, this investigation quantified baseline social isolation across three dimensions—family isolation, friend isolation, and subjective isolation—alongside cognitive function scores of older adults, measured across all three waves. Through latent class growth models, heterogeneous cognitive trajectories were delineated. The influence of family isolation, friend isolation, and subjective isolation on these cognitive trajectories was examined employing multinomial logistic regression analysis.

**Results:**

The study included 6,378 participants aged 60 and above, revealing three primary cognitive trajectories: High baseline stable group (68.8%), High baseline but declining group (21.7%), and Low baseline deteriorating group (9.5%). Adjusting for variables such as personal physical characteristics, social networks, living and working conditions, and the surrounding policy environment, the findings indicated that family isolation did not significantly affect cognitive function’s high-level decline or low-level deterioration. Conversely, friend isolation markedly increased the risk of high-level cognitive decline (OR = 1.289) and low-level cognitive deterioration (OR = 1.592). Similarly, subjective isolation significantly heightened the risk for both high-level decline (OR = 1.254) and low-level deterioration (OR = 1.29) in cognitive function.

**Conclusion:**

Mitigating friend and subjective isolation among older adults appears to be a more effective strategy in preventing or delaying cognitive decline, potentially reducing the strain on healthcare and social welfare systems.

## 1 Introduction

Cognitive function refers to the brain’s ability to retrieve, process, transform, and store information (1). A decline in cognitive function that exceeds what is typically observed as part of the normal aging process may result in cognitive impairments and ultimately lead to dementia (2). Severe cognitive impairment can significantly diminish the quality of life for older individuals (3, 4). These impairments are associated with an increased risk of long-term hospitalization (5) and elevated mortality rates among older adults (6). Currently, the global population of older adults with dementia is approximately 50 million, a figure projected to rise to 152 million by 2050 due to demographic aging (7). Notably, China accounts for about 25% of the global dementia population, posing a significant challenge for policymakers, healthcare professionals, and families (8, 9). The recent implementation of social distancing measures during the COVID-19 pandemic has intensified concerns regarding social isolation and its potential health consequences (10, 11). Despite increased interest, the relationship between social isolation and cognitive health in older adults remains unclear, with current literature presenting conflicting findings (12, 13). This underscores the necessity for further investigation into this complex relationship.

Social isolation can be categorized into objective and subjective dimensions. Objective isolation is measurable by the diminished size of social networks and reduced frequency of social interactions (14). In China, older adults significantly value personal and familial connections, necessitating the subdivision of objective isolation into two distinct categories: family isolation and friend isolation (15). Subjective isolation, on the other hand, pertains to an individual’s perception of the discrepancy between the desired and actual quantity and quality of social interactions (16). Examination of the interplay between the two facets of social isolation has highlighted a synergistic effect on health outcomes, affecting various health metrics differently (17, 18). Accordingly, this study aims to examine both the objective and subjective aspects of social isolation, with a specific focus on distinguishing between family and friend isolation. This approach endeavors to offer a more holistic understanding and deeper insight into the dynamics between social isolation and cognitive function among older adults.

Changes in cognitive function represent a long-term and dynamic process, which necessitates detailed examination through longitudinal studies. However, majority of existing longitudinal research on this topic is conducted in Western countries, often neglecting the intricate interplay between social structures and individual behaviors characteristic of Eastern collectivist cultures. Compared to Western contexts, there is a notable dearth of longitudinal studies in Eastern settings exploring the relationship between social isolation and cognitive function among older adults, with much of the research limited to cross-sectional studies at a single time point. Furthermore, from a life course perspective, the development of cognitive function is not only dynamic and heterogeneous but is also profoundly influenced by social interactions and relationships throughout an individual’s life. This perspective enables an exploration of how isolation from family and friends impacts cognitive trajectories among older adults (19). Nevertheless, most longitudinal studies rely on traditional regression or growth curve modeling techniques, which are limited to identifying average associations and fail to account for the variability in cognitive trajectories among different older adults (20, 21).

Hence, this research endeavors to conduct ongoing assessments of cognitive function within a nationally representative cohort of Chinese older adults, employing Latent Class Growth Modeling (LCGM) to delineate heterogeneous cognitive trajectories in this population. The primary aim is to scrutinize the associations between objective isolation (including both family and friend isolation), subjective isolation and heterogeneous cognitive trajectories, all within the unique milieu of an Eastern collectivist culture. Through these investigations, the study aspires to offer evidence-based insights for the formulation of precise cognitive health enhancement strategies tailored to older adults in Eastern collectivist societies, which can contribute significantly to the prevention of dementia and related conditions, decelerate cognitive aging processes, and thereby reduce the pressures on healthcare and social welfare systems.

## 2 Methods

### 2.1 Data source and sample selection

This investigation relies on data from the China Longitudinal Aging Social Survey (CLASS), employing a stratified multi-stage probability sampling design to select individuals aged 60 and above from 28 provincial units across the country. The CLASS dataset includes detailed records on personal demographics, physical and mental health, and social support systems pertinent to older adults. Initiated in 2014, this study included biennial follow-up surveys, with the most recent conducted in 2020. The study leverages data from the 2016, 2018, and 2020 waves. From 2016 onwards, a total of 11,471 older adults had contributed data to CLASS.

**Figure 1:**
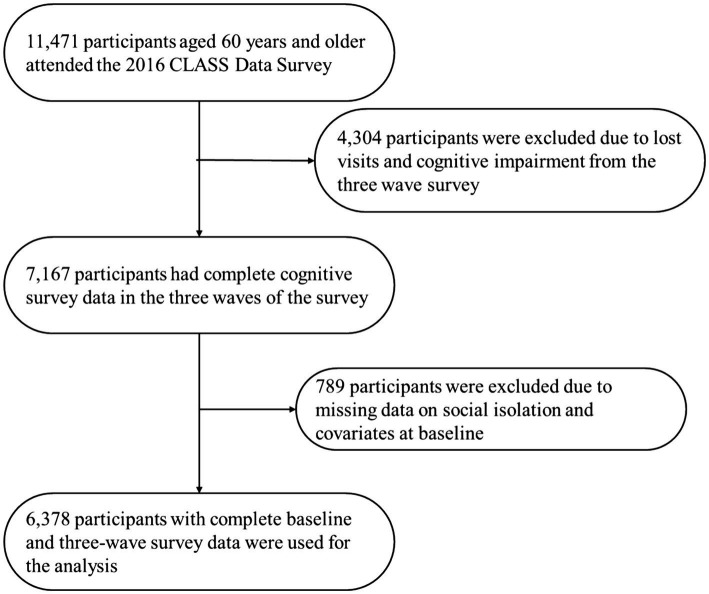
Flow chart of the CLASS.

Given the inevitable attrition in longitudinal surveys, and recognizing that a prerequisite for completing the psychological assessment scale is that the participants do not exhibit signs of cognitive impairment—specifically, a cognitive score of no less than 5 points (22)—this study excludes 4,304 older adults who were either lost to follow-up or demonstrated cognitive impairment across the three waves of surveys conducted from 2016 to 2020. This research aims to analyze the influence of the baseline data from 2016 on the heterogeneous trajectories of cognitive development among older adults. To ensure data completeness and accuracy, cases with missing information on multidimensional social isolation and other essential covariates at the baseline stage were eliminated, resulting in a final sample size of 6,378 individuals. The flow chart of the CLASS follow-up and the sample selection of the current analysis is presented in Figure 1.

### 2.2 Measurements

#### 2.2.1 Social isolation

To assess objective isolation, this study employed the Lubben Social Network Scale (LSNS) (23), as developed by Lubben and colleagues. This instrument evaluates the extent of social engagement among older adults by quantifying the number of family members and friends available for private discussions and support. It comprises two distinct components: family isolation (Cronbach’s alpha = 0.812) and friend isolation (Cronbach’s alpha = 0.856), incorporating a total of six questions. Response options range from 0 (none), 1 (one), 2 (two), 3 (three to four), 4 (five to eight), to 5 (nine or more). An aggregate score for family or friend networks below 6 points (23) signifies isolation in that domain, coded as 1 for isolated and 0 for not isolated.

Subjective isolation is quantified through the loneliness variable (24), which is evaluated using a single item from the Center for Epidemiologic Studies Depression Scale (CES-D): “How often did you feel lonely in the past week?” (25). Responses to this item are scored on a three-point scale: 1 (never), 2 (sometimes), and 3 (often). Participants who respond with “2” or “3” are classified as experiencing subjective isolation and are assigned a code of 1, while those who respond with “1” are coded as 0, indicating the absence of subjective isolation.

#### 2.2.2 Cognitive function

The evaluation of cognitive function within this study utilizes the Mini-mental State Examination (MMSE) as provided by CLASS (26). This instrument measures the cognitive performance of older adults across various domains: orientation (5 items), memory (3 items), attention and calculation (5 items), and recall (3 items), including a total of 16 questions. Responses are scored on a binary scale: 1 point for each correct answer and 0 points for each incorrect answer, resulting in a possible score range of 0 to 16 points. A higher total score is indicative of superior cognitive functioning.

#### 2.2.3 Covariates

In this study, covariates were selected based on the ecological model of health (27), examining determinants of cognitive function across various strata to underpin cognitive impairment prevention strategies among older adults. The specific covariates are as follows: The model’s innermost layer considers individual physical characteristics, including age (>60 years), gender (female, male), the presence of chronic diseases (yes or no), depression (assessed via the Center for Epidemiological Studies Depression Scale (CES-D) (25), with a total score of 24 points where higher scores denote more severe depression), and basic Activities of Daily Living (ADL) status (scored out of 33 points across 11 activities, with higher scores reflecting decreased basic activity capability). Notably, since subjective isolation is measured using an item from the CES-D, the depression variable in this analysis excludes this specific item to avoid overlap. The model’s second layer focuses on social networks, highlighting marital status (married, unmarried/divorced/widowed). The third layer explores living and working conditions, delineated by educational level (illiterate, primary school, junior high school, high school and above), employment status (employed, not employed), and household registration (rural, urban). The outermost layer assesses the policy environment, specifically the presence of pension insurance (having at least one type of pension insurance, none).

### 2.3 Statistical methods

LCGM is a comprehensive mixed model that combines the characteristics of Latent Growth Curve Modeling (LGCM) and Latent Class Analysis (20, 21, 28). First, it describes the trajectory of the growth curve using the latent growth curve model, and then further refines the analysis by determining the number of latent trajectory classes, categorizing individuals into distinct latent groups based on their growth trajectories. This method allows for a detailed examination of the variability in cognitive function among older adults, facilitating a deeper understanding of the heterogeneity within this demographic.

In determining the optimal number of latent trajectory classes, our approach involved incrementally increasing the number of classes until the improvement in model performance ceased to be significant. The evaluation and selection of the final model were guided by various statistical metrics: Sample-size Adjusted Bayesian Information Criterion (SABIC), Entropy, Bootstrap Likelihood Ratio Test (BLRT), and Vuong-Lo–Mendell–Rubin Likelihood Ratio Test (VLMR-LRT). An appreciable decrease in SABIC signals an enhanced fit of the model. Entropy values, which range from 0 to 1, reflect the clarity of class distinction, with higher scores indicating more distinct class separations. Both VLMR-LRT and BLRT compare models with varying numbers of classes, where a significant *p*-value supports the model with a greater number of classes as providing a superior fit. Additionally, it is essential for model validity that each identified class comprises at least 5% of the sample size. In the selection of the ultimate model, the interpretability and meaningfulness of each trajectory class also play a pivotal role.

Firstly, employing LCGM enables the precise differentiation of diverse cognitive trajectories among groups, enhancing the accuracy of identifying distinct cognitive patterns. Secondly, between-class differences were examined by Kruskal-Wallis rank sum test for continuous variables or *χ*^2^ test for categorical variables. Lastly, the application of multinomial logistic regression analysis aids in uncovering the impact of the baseline data from 2016 on the heterogeneous cognitive development trajectories among older adults. This approach provides empirical support for the detection and intervention of cognitive impairments in older adults. The analysis leverages Mplus8.3 software for identifying the heterogeneous cognitive trajectories among older adults, while Stata 17.0 is used for conducting *χ*^2^/t-tests and multinomial logistic regression analysis, with a significance level set at *p* < 0.05.

## 3 Results

### 3.1 Heterogeneous cognitive trajectories of older adults

LCGM was employed to identify heterogeneous cognitive trajectories among older adults, with the analysis guided by key statistical metrics as detailed in Table 1. The investigation resulted in a 3-class LCGM model that balanced simplicity with adequate fit and clear classification (Entropy value = 0.898). Although a model with more classes showed some improvements in specific indicators, it was deemed potentially susceptible to overfitting. Therefore, the 3-class model was chosen for its statistical robustness, simplicity, and ability to effectively capture the underlying heterogeneity within the data.

**Table 1:** Fit statistics for cognitive trajectories aged 60 years and older adults from CLASS.

Fit statistic	Number of classes					
	1	2	3	4	5	6
AIC^a^	90106.022	89203.560	88048.480	86218.510	85625.017	84651.300
aBIC^a^	90134.685	89232.220	88087.890	86268.670	85685.925	84722.960
Entropy^b^		0.861	0.898	0.884	0.886	0.906
LMR^c^		*p* < 0.001	*p* < 0.001	*p* < 0.001	*p* < 0.001	*p* < 0.001
BLRT^c^		*p* < 0.001	*p* < 0.001	*p* < 0.001	*p* < 0.001	*p* < 0.001
Smallest class^d^		20.54%	9.52%	8.22%	5.46%	4.41%

CLASS, China Longitudinal Aging Social Survey; AIC, Akaike’s information criterion; aBIC, Bayesian information criteria for sample size adjustment; LMR, lo–mendell–rubin; BLRT, Bootstrap likelihood ratio test.

a A lower absolute value suggests a better model fit.

b Range from 0 to 1, higher scores indicating more distinct class separations.

c Significant *p*-values indicate that models containing more categories fit better.

d No less than 5% of total count in a class.

**Figure 2:**
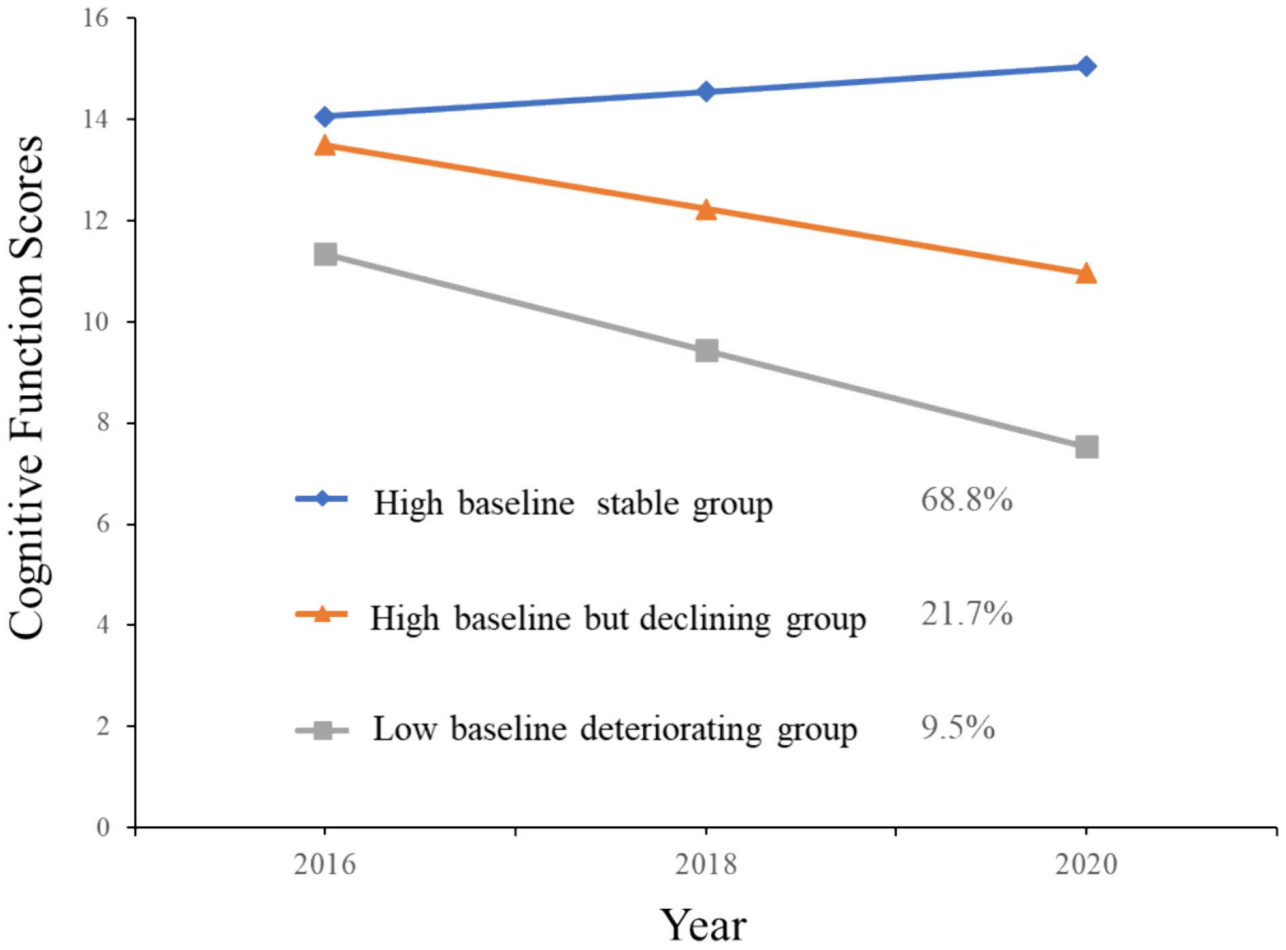
Trajectories of cognitive function scores by increasing year among older adults.

The analysis delineated three heterogeneous cognitive trajectories, with each class’s intercepts, slopes, and corresponding statistical tests summarized in Table 2 and illustrated in Figure 2. The first trajectory, including 68.8% of the sample (*n* = 4,388), displayed the highest average cognitive function at baseline (Intercept *I* = 14.055, *p* < 0.001) with a stable, albeit slight, upward trajectory (Slope *S* = 0.563, *p* < 0.001), and was thus classified as the “High baseline stable group.” The second trajectory, comprising 21.7% of the sample (*n* = 1,386), started with a relatively high baseline cognitive function (Intercept *I* = 13.499, *p* < 0.001) but experienced a significant decline over time (Slope *S* = −1.268, *p* < 0.001), leading to its classification as the “High baseline but declining group.” The third trajectory, accounting for 9.5% of participants (*n* = 604), began with the lowest average cognitive function (Intercept *I* = 11.341, *p* < 0.001) and underwent a more rapid decline (Slope *S* = −1.901, *p* < 0.001), identifying this group as the “Low baseline deteriorating group.”

**Table 2:** The final three-group trajectory model of cognitive function of aged 60 years and older adults from CLASS.

Trajectory group	*n* (%)	Intercept			Linear slope		
		Est.	SE	*p* value	Est.	SE	*p* value
High baseline stable group	4,388 (68.8%)	14.055	0.042	<0.001	0.563	0.024	<0.001
High baseline but declining group	1,386 (21.7%)	13.499	0.101	<0.001	−1.268	0.087	<0.001
Low baseline deteriorating group	604 (9.5%)	11.341	0.27	<0.001	−1.901	0.117	<0.001

CLASS, China Longitudinal Aging Social Survey; Est., parameter estimate; SE, standard error of parameter estimate.

### 3.2 Descriptive statistical analysis of study subjects

Table 3 presents the status of multidimensional social isolation among older adults. Out of 6,378 individuals, the prevalence rates of family isolation, friend isolation, and subjective isolation were 19.18, 28.77, and 42.79%, respectively. Additionally, it is important to note that 6.7% of the participants experienced both objective and subjective isolation concurrently. Comparative analyses across different cognitive trajectories highlighted that people in the “Low baseline deteriorating group” exhibited higher rates of family isolation, friend isolation, and subjective isolation than those in the “High baseline stable group,” with these differences reaching statistical significance (*p* < 0.01).

**Table 3:** Baseline characteristics of the participants according to trajectories of cognitive function aged 60 years and older adults from CLASS.

Characteristic	*n* (%)	Trajectory of cognitive function			*p* value
		High baseline stable group	High baseline but declining group	Low baseline deteriorating group	
Observations	6,378	4,388 (68.8)	1,386 (21.7)	604 (9.5)	
Social isolation					
Family isolation					
No	5,155 (80.82)	3,656 (82.01)	1,028 (78.29)	471 (77.59)	0.001**
Yes	1,223 (19.18)	802 (17.99)	285 (21.71)	136 (22.41)	
Friend isolation					
No	4,543 (71.23)	3,275 (73.46)	884 (67.33)	384 (63.26)	0.000***
Yes	1,835 (28.77)	1,183 (26.54)	429 (32.67)	223 (36.74)	
Subjective isolation					
No	3,649 (57.21)	2,647 (59.38)	704 (53.62)	298 (49.09)	0.000***
Yes	2,729 (42.79)	1,811 (40.62)	609 (46.38)	309 (50.91)	
Physical characteristics					
Gender					
Female	3,107 (48.71)	2,108 (47.29)	679 (51.71)	320 (52.72)	0.002**
Male	3,271 (51.29)	2,350 (52.71)	634 (48.29)	287 (47.28)	
Age					
60–74	5,386 (84.45)	3,969 (89.03)	1,046 (79.66)	371 (61.12)	0.000***
75–85	933 (14.63)	467 (10.48)	250 (19.04)	216 (35.58)	
85+	59 (0.93)	22 (0.49)	17 (1.29)	20 (3.29)	
Chronic diseases					
No	2,823 (44.26)	1,987 (44.57)	593 (45.16)	243 (40.03)	0.082
Yes	3,555 (55.74)	2,471 (55.43)	720 (54.84)	364 (59.97)	
ADL^a^	11.38 ± 1.40	11.31 ± 1.27	11.43 ± 1.44	11.77 ± 2.04	0.000***
Depression	13.81 ± 2.73	13.74 ± 2.71	13.79 ± 2.73	14.32 ± 2.81	0.000***
Social networks					
Marital status					
Single^b^	1,467 (23.00)	918 (20.59)	345 (26.28)	204 (33.61)	0.000***
Married	4,911 (77.00)	3,540 (79.41)	968 (73.72)	403 (66.39)	
Living and working conditions					
Educational level					
Illiterate	1,462 (22.92)	812 (18.21)	401 (30.54)	249 (41.02)	0.000***
Primary school	2,672 (41.89)	1,872 (41.99)	542 (41.28)	258 (42.50)	
Junior school	1,647 (25.82)	1,290 (28.94)	276 (21.02)	81 (13.34)	
High school and above	597 (9.36)	484 (10.86)	94 (7.16)	19 (3.13)	
Employment status					
Not employed	5,488 (86.05)	3,788 (84.97)	1,151 (87.66)	549 (90.44)	0.000***
Employed	890 (13.95)	670 (15.03)	162 (12.34)	58 (9.56)	
Residence					
Rural	3,180 (49.86)	2,124 (47.64)	673 (51.26)	383 (63.10)	0.000***
Urban	3,198 (50.14)	2,334 (52.36)	640 (48.74)	224 (36.90)	
Policy environment					
Pension insurance					
No	1,677 (26.29)	1,121 (25.15)	375 (28.56)	181 (29.82)	0.006**
Yes	4,701 (73.71)	3,337 (74.85)	938 (71.44)	426 (70.18)	

*, **, and *** indicate significance at the levels of 0.05, 0.01, and 0.001, respectively.

Data are means ± SD or *n* (%). Between-class differences were examined by Kruskal-Wallis rank sum test for continuous variables or *χ*^2^ test for categorical variables.

a ADL, activities of daily living.

b Single (unmarried, divorced, widowed).

Demographically, males represented a slight majority (51.29%), and a significant portion of the sample fell within the 60–74 age range (84.45%). The presence of chronic diseases was common (55.74%), the average ADL score was 11.38 ± 1.40, and the average depression score stood at 13.81 ± 2.73, reflecting the health status of older adults. Our findings indicate that males are more likely to experience family and friend isolation, whereas females are more prone to subjective isolation. With increasing age, the proportion experiencing objective isolation gradually decreases, while the proportion experiencing subjective isolation increases. Higher ADL and depression scores are associated with a greater likelihood of subjective isolation. In terms of social networks, most of the sample were married individuals (77.00%), with a nearly equal distribution between rural (49.86%) and urban (50.14%) residences. The study showed that unmarried and rural older adults are more susceptible to subjective isolation. Regarding educational and occupational background, the majority of the sample had an education level of primary school or below (41.89%), with only 9.36% having attained high school education or higher. Most older adults were not employed (86.05%), with a significant portion being retired. The findings suggest that retired older adults are more likely to experience subjective isolation. In the policy environment, a large majority had some form of pension insurance (73.71%). However, older adults without pension insurance were found to be more vulnerable to both subjective and objective isolation.

Further Kruskal-Wallis rank sum test or *χ*^2^ test comparing characteristics among cognitive groups revealed that the “Low baseline deteriorating group” was more likely to include older females with more severe depression and diminished ADL capabilities. Social network analyses indicated higher proportions of unmarried individuals and those residing in rural areas within this group. Concerning living and working conditions, this group had lower educational levels and a higher percentage of non-working individuals. From a policy perspective, a lack of pension insurance was more prevalent among those in the “Low baseline deteriorating group” compared to the “High baseline stable group.”

### 3.3 Impact of multidimensional social isolation on heterogeneous cognitive trajectories among older adults

The analysis employed a multinomial logistic regression model, designating the identified cognitive trajectory groups (High baseline stable group, High baseline but declining group, Low baseline deteriorating group) as the dependent variable, with the High baseline stable group serving as the reference category. This model assessed the influence of multidimensional social isolation, alongside various covariates such as physical characteristics, social networks, living and working conditions, and policy environment, on cognitive trajectories among older adults. The findings, detailed in Table 4, primarily focused on the effects of different facets of social isolation.

**Table 4:** Multinomial logistic regression analysis for the association between multidimensional social isolation and heterogeneous cognitive trajectories among older adults.

Characteristic	High baseline but declining group (Ref: High baseline stable group)			Low baseline deteriorating group (Ref: High baseline stable group)		
	OR	95% CI	*p*	OR	95%	*p*
Social isolation						
Family isolation						
No	Ref.	Ref.	Ref.	Ref.	Ref.	Ref.
Yes	1.111	0.930 ~ 1.327	0.245	1.075	0.838 ~ 1.380	0.568
Friend isolation						
No	Ref.	Ref.	Ref.	Ref.	Ref.	Ref.
Yes	1.289	1.103 ~ 1.506	0.001	1.592	1.282 ~ 1.978	0.000
Subjective isolation						
No	Ref.	Ref.	Ref.	Ref.	Ref.	Ref.
Yes	1.254	1.095 ~ 1.436	0.001	1.29	1.065 ~ 1.563	0.009
Physical characteristics						
Gender						
Female	Ref.	Ref.	Ref.	Ref.	Ref.	Ref.
Male	0.913	0.801 ~ 1.040	0.169	0.93	0.772 ~ 1.121	0.446
Age						
60–74	Ref.	Ref.	Ref.	Ref.	Ref.	Ref.
75–85	1.859	1.558 ~ 2.218	0.000	4.335	3.515 ~ 5.346	0.000
>85	2.522	1.314 ~ 4.840	0.005	7.651	3.986 ~ 14.686	0.000
Chronic diseases						
No	Ref.	Ref.	Ref.	Ref.	Ref.	Ref.
Yes	0.929	0.817 ~ 1.057	0.264	0.981	0.814 ~ 1.183	0.844
ADL^a^	1.034	0.988 ~ 1.082	0.151	1.094	1.042 ~ 1.149	0.000
Depression	0.978	0.953 ~ 1.002	0.076	1.021	0.984 ~ 1.058	0.272
Social network						
Marital status						
Single^b^	Ref.	Ref.	Ref.	Ref.	Ref.	Ref.
Married	0.918	0.787 ~ 1.071	0.278	0.861	0.700 ~ 1.059	0.156
Living and working conditions						
Educational level						
Illiterate	Ref.	Ref.	Ref.	Ref.	Ref.	Ref.
Primary school	0.624	0.533 ~ 0.731	0.000	0.55	0.448 ~ 0.675	0.000
Middle school	0.486	0.402 ~ 0.587	0.000	0.322	0.242 ~ 0.428	0.000
High school and above	0.416	0.319 ~ 0.541	0.000	0.186	0.113 ~ 0.306	0.000
Employment status						
Not employed	Ref.	Ref.	Ref.	Ref.	Ref.	Ref.
Employed	0.827	0.685 ~ 0.999	0.048	0.696	0.518 ~ 0.934	0.016
Residence						
Rural	Ref.	Ref.	Ref.	Ref.	Ref.	Ref.
Urban	1.002	0.873 ~ 1.151	0.973	0.633	0.518 ~ 0.773	0.000
Policy environment						
Pension insurance						
No	Ref.	Ref.	Ref.	Ref.	Ref.	Ref.
Yes	0.916	0.792 ~ 1.059	0.235	0.991	0.808 ~ 1.215	0.928
Constant	0.414	0.219 ~ 0.781	0.006	0.054	0.025 ~ 0.117	0.000

Ref, reference; OR, odds ratio; 95% CI, 95% confidence intervals.

a ADL, activities of daily living.

b Single (unmarried, divorced, widowed).

**Figure 3:**
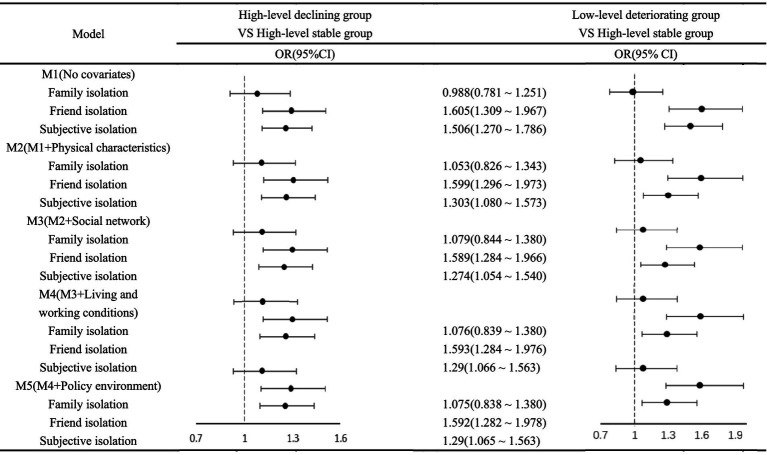
Stratified analysis for association between multidimensional social isolation and heterogeneous cognitive trajectories among older adults.

The analysis revealed that family isolation did not significantly influence the risk of transitioning to the High baseline but declining group or Low baseline deteriorating group cognitive groups. Conversely, friend isolation markedly heightened the risk of high-level cognitive declining (OR = 1.289) and low-level cognitive deteriorating (OR = 1.592). Similarly, subjective isolation significantly elevated the risks for high-level cognitive declining (OR = 1.254) and Low baseline deteriorating group (OR = 1.29). The inclusion of covariates such as individual physical characteristics, social networks, living and working conditions, and policy environment, resulted in only minimal alterations to the original findings regarding the influence of social isolation on cognitive trajectories, suggesting a strong independent effect of social isolation factors. The detailed outcomes of this extended analysis are visually presented in Figure 3.

Examining the covariates, older age brackets (>85 years and 75–84 years) were significantly associated with increased risks of High baseline but declining group (OR = 2.522 and 1.859, respectively) and Low baseline deteriorating group (OR = 7.651 and 4.335, respectively) in cognitive function. Higher ADL scores were notably linked to an increased risk of cognitive function’s Low baseline deteriorating group (OR = 1.904). Urban household registration was associated with a reduced risk of cognitive function’s Low baseline deteriorating group (OR = 0.633). Higher educational levels and being employed were protective against the risk of high-level cognitive declining and Low baseline deteriorating group, with significant OR values indicating a reduced risk across these categories. These findings highlight the complex interplay of social isolation and other factors in influencing the cognitive aging process among older adults, underscoring the significance of multidimensional social support and engagement in maintaining cognitive health.

### 3.4 Sensitivity analysis

**Figure 4:**
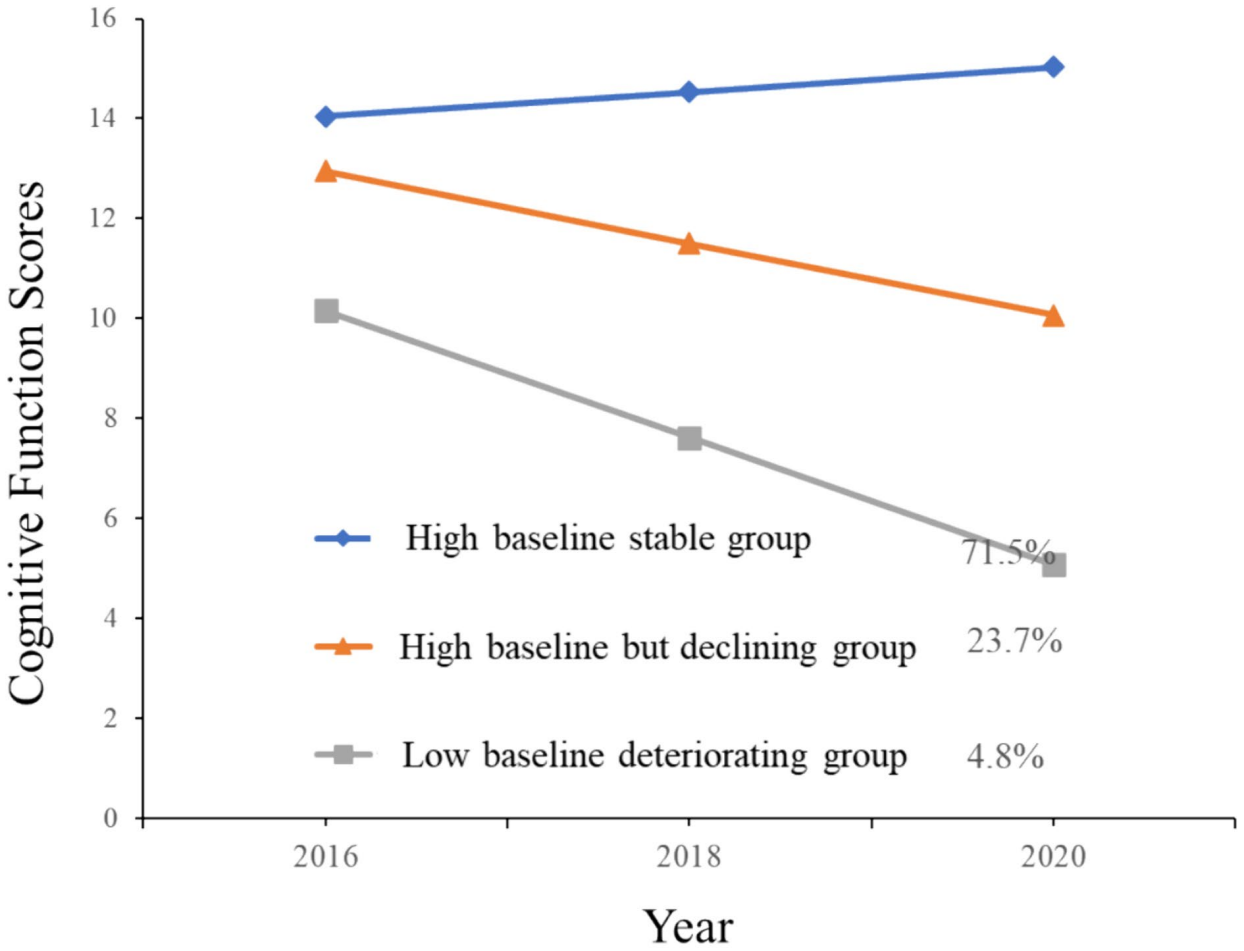
Trajectories of cognitive function scores by increasing year among older adults (including cognitively impaired groups in 2018 and 2020).

To verify the robustness of our research findings, we implemented two measures: First, we lowered the sample selection criteria to include older adults with cognitive impairments identified in 2018 and 2020, thereby comprehensively considering the cognitive trajectories of older adults with lower cognitive health levels. Figure 4 illustrates these results. After relaxing the sample selection criteria, the final sample size reached 6,499 older adults. The analysis results indicate that, despite the more inclusive sample criteria, the overall conclusions regarding the cognitive trajectories of older adults remained consistent with the original analysis. This consistency suggests strong support for the reliability of our classification of cognitive trajectories among older adults.

Secondly, we altered the treatment of the independent variables by analyzing social isolation as continuous variables rather than dichotomous ones. To avoid the potential information loss that occurs when continuous variables are converted to binary categories, we conducted an original treatment of the three dimensions of social isolation. Specifically, the dimensions of family isolation and friend isolation were converted into continuous variables of family networks and friend networks (where higher scores indicate a greater number of family members and friends the older adults can meet, contact, discuss private matters with, and seek help from). Subjective isolation retained the three-point scoring system of “1 (none), 2 (sometimes), 3 (often).” Table 5 presents these results. The findings show that a robust friend network significantly reduces the risk of both high-level cognitive decline and low-level cognitive deterioration, while subjective isolation significantly increases the risk of high-level cognitive decline. These results are consistent with our previous conclusions, further validating the relationship between social isolation and cognitive trajectories.

**Table 5:** Multinomial logistic regression analysis for the association between multidimensional social isolation and heterogeneous cognitive trajectories among older adults (analyzing the raw data for multidimensional social isolation as continuous variables).

Characteristic	High baseline but declining group (Ref: High baseline stable group)			Low baseline deteriorating group (Ref: High baseline stable group)		
	OR	95% CI	*p*	OR	95% CI	*p*
Social isolation						
Family isolation	0.975	0.948 ~ 1.002	0.072	1.003	0.947 ~ 1.063	0.910
Friend network	0.962	0.939 ~ 0.987	0.002	0.956	0.909 ~ 1.006	0.082
Subjective isolation	1.172	1.059 ~ 1.298	0.002	1.138	0.922 ~ 1.406	0.228
Physical characteristics						
Gender						
Female	Ref.	Ref.	Ref.	Ref.	Ref.	Ref.
Male	0.925	0.818 ~ 1.045	0.211	1.049	0.804 ~ 1.367	0.727
Age						
60–74	Ref.	Ref.	Ref.	Ref.	Ref.	Ref.
75–85	2.253	1.919 ~ 2.645	0.000	8.505	6.445 ~ 11.224	0.000
>85	2.838	1.577 ~ 5.109	0.001	20.101	10.127 ~ 39.898	0.000
Chronic diseases						
No	Ref.	Ref.	Ref.	Ref.	Ref.	Ref.
Yes	0.954	0.844 ~ 1.077	0.446	1.163	0.887 ~ 1.526	0.275
ADL^a^	1.053	1.010 ~ 1.097	0.015	1.157	1.095 ~ 1.221	0.000
Depression	0.984	0.960 ~ 1.008	0.182	1.042	0.988 ~ 1.099	0.13
Social network						
Marital status						
Single^b^	Ref.	Ref.	Ref.	Ref.	Ref.	Ref.
Married	0.895	0.775 ~ 1.033	0.13	0.832	0.626 ~ 1.106	0.206
Living and working conditions						
Education level						
Illiterate	Ref.	Ref.	Ref.	Ref.	Ref.	Ref.
Primary school	0.557	0.481 ~ 0.644	0.000	0.386	0.290 ~ 0.514	0.000
Middle school	0.422	0.353 ~ 0.504	0.000	0.26	0.169 ~ 0.400	0.000
High school and above	0.314	0.241 ~ 0.409	0.000	0.183	0.090 ~ 0.373	0.000
Employment status						
Unemployed	Ref.	Ref.	Ref.	Ref.	Ref.	Ref.
Employed	0.733	0.610 ~ 0.881	0.001	0.737	0.475 ~ 1.144	0.174
Residence						
Rural	Ref.	Ref.	Ref.	Ref.	Ref.	Ref.
Urban	0.883	0.775 ~ 1.007	0.063	0.434	0.323 ~ 0.582	0.000
Policy environment						
Pension insurance						
No	Ref.	Ref.	Ref.	Ref.	Ref.	Ref.
Yes	0.941	0.820 ~ 1.079	0.383	0.966	0.725 ~ 1.287	0.815
Constant	1.116	0.543 ~ 2.293	0.765	0.019	0.005 ~ 0.071	0.000

Ref, reference; OR, odds ratio; 95% CI, 95% confidence intervals.

a ADL, activities of daily living.

b Single (unmarried, divorced, widowed).

## 4 Discussion

This investigation delves into the nuances of how family, friend, and subjective isolation uniquely influence cognitive trajectories among older adults in China. Utilizing latent class growth models on data from three successive waves of the Chinese Longitudinal Aging Social Survey (CLASS) spanning 2016 to 2020, with a sample of 6,378 participants aged 60 and above, we identified three cognitive trajectories: High baseline stable group, High baseline but declining group, and Low baseline deteriorating group. Our findings reveal that while family isolation has no significant impact, friend and subjective isolation markedly increase the risk of cognitive decline. By distinguishing between types of social isolation, this study enhances our understanding of their distinct effects on cognitive aging, informing more effective public health strategies for older adults.

This study reveals that 19.18% of older adults experience family isolation, 28.77% friend isolation, and 42.79% subjective isolation. These statistics align with prior research indicating objective isolation rates between 18.4 and 30.5% (29–31) and subjective isolation around 43% (24, 32). Given that a substantial portion of this study’s participants (84.45%) were aged 60–74 years, primarily younger older adults, the observed rates of social isolation were comparatively lower. Among these, older adults experience less isolation within families than with friends, which could be attributed to the strong caregiving culture in China, where familial support, especially from younger generations, plays a crucial role in older adult care (33).

Through the LCGM approach, three distinct cognitive trajectories among older adults were identified: High baseline stable group (68.8%), High baseline but declining group (21.7%), and Low baseline deteriorating group (9.5%). These categories correspond with findings from other studies (34, 35), although proportions vary, likely due to different study criteria and the inclusion of a younger senior population in our research. The significant representation of the High baseline stable group might be explained by the exclusion of individuals with cognitive impairments and the predominance of younger y older adults. Furthermore, variations in follow-up duration across studies might contribute to differing observations of cognitive decline (29, 36). Notably, while some research identifies diverse cognitive trajectories (29, 37), the number of trajectories detected often hinges on the interplay between subjective judgment and objective criteria in determining the optimal number of classes (28). This study’s findings underscore the complex dynamics of social isolation and cognitive health, highlighting the need for targeted interventions to mitigate cognitive decline among older adults.

Our findings reveal that while family isolation does not significantly affect cognitive decline at high levels or lead to low-level deterioration, both friend isolation and subjective isolation are notable risk factors for these adverse outcomes. This observation is consistent with existing literature, which has documented the detrimental effects of social isolation on cognitive functions (4, 24, 38, 39). Specifically, friend and subjective isolations are implicated in fostering adverse health behaviors (40, 41) that are linked to cognitive decline (36, 42), highlighting their potential roles in triggering these negative outcomes. However, friend isolation may predominantly affect cognitive abilities by reducing social stimulation, whereas subjective isolation could impact cognitive functions more through internal psychological stress. Frequent social interactions can bolster or maintain cognitive reserves, enhancing cognitive performance of older adults through the reallocation of brain networks or the adoption of alternative cognitive strategies (38, 43). Regular social engagement also stimulates cognitive activity, potentially increasing cortical synaptic density and, thereby, supporting the preservation of cognitive function in older age (4, 44). On the other hand, subjective isolation acts through psychological mechanisms, serving as a stressor that may lead to the overactivation of the hypothalamic–pituitary–adrenal (HPA) axis and increased expression of pro-inflammatory factors. These physiological responses can induce neurodegenerative changes in the hippocampus, a crucial area for memory and cognitive functions (45, 46). Moreover, subjective isolation can cause older adults to internalize their focus on social needs and negative emotions (40), which can consume substantial cognitive resources and negatively impact the execution of cognitive functions. This comprehensive understanding underscores the importance of addressing both friend and subjective isolations in efforts to mitigate cognitive decline among older adults.

Compared to friend isolation, this study found that family isolation does not significantly affect high-level cognitive decline and low-level deterioration in cognitive function, which aligns with some previous research findings (47, 48). This discrepancy can be attributed to several key factors. First, distinct functional roles. Family members predominantly fulfill economic and caregiving needs of older adults, while friends contribute to recreational and social developmental needs of older adults (44, 48). Friends provide diverse social stimuli, crucial for maintaining cognitive function, offering a range of social interactions that can stimulate cognitive processes in ways family relationships might not. Second, nature of interactions. Family interactions often carry obligatory undertones and may be more prone to negative exchanges, potentially causing greater psychological stress or harm to older adults (49, 50). Conversely, friendships, grounded in shared interests, tend to foster positive interactions, thereby enhancing sense of social identity and psychological well-being among older adults (47, 51). This positive dynamic with friends plays a crucial role in cognitive health maintenance. Third, mediation by friend networks. Functional support from family is pivotal for older adults to broaden their friend networks. The influence of family isolation on cognitive function is potentially fully mediated by friend isolation (48), indicating that the quality and extent of friendships are critical determinants of cognitive health. This mediation is particularly relevant in cultures where familial ties play a central role in social structures. Furthermore, some studies suggest no significant correlation between social isolation and cognitive trajectories, possibly due to difficulties in measuring and quantifying such complex social phenomena (13, 52, 53). The lack of a standardized tool for assessing social isolation contributes to these varying outcomes. Additionally, the hypothesis of a reverse causal relationship between social isolation and cognitive decline suggests that social isolation might be a consequence, rather than a cause, of cognitive deterioration (13, 36). This hypothesis underscores the importance of conducting longitudinal studies to better understand the directionalities and nuances of the relationship between social isolation and cognitive health in older adults.

Given the significant risk that friend isolation and subjective isolation pose to older adults in terms of high-level decline and low-level deterioration, it is crucial to implement measures to mitigate these impacts. A national strategy tailored to Chinese social and cultural norms, similar to the UK’s Anti-Social Isolation strategy (24), could boost social engagement using digital platforms, targeting those in remote areas. Additionally, enhancing public infrastructure and fostering family and community support can further facilitate social interactions among older adults. Early identification and personalized interventions for cognitive decline should also be considered to create a supportive ecosystem for older adults.

While this study provides valuable insights into the intricate dynamics between social isolation and cognitive trajectories among older adults, it is imperative to recognize certain limitations that could influence the generalizability of the findings. Firstly, the study relied on data from a broad social survey, employing relatively simplified tools for assessing complex constructs like subjective isolation, which was gauged with a single-item measure. This approach might not fully encapsulate the nuanced facets of loneliness. Moreover, cognitive function was assessed using the MMSE, which may have limitations in detecting subtle cognitive changes, particularly in early stages of impairment due to its ceiling effects. Secondly, the use of self-reported questionnaires, while practical for large-scale data collection, introduces potential biases related to participants’ memory accuracy, understanding of questions, and response tendencies, which might affect the data’s objectivity and precision. Thirdly, the longitudinal nature of the study led to exclusion of individuals who were less healthy, had lower education levels, or exhibited poorer cognitive function due to attrition or incomplete data. This could result in selection bias, possibly overlooking those with consistently low cognitive functioning. Finally, the relatively brief duration of follow-up might not be sufficient to capture the gradual progression of cognitive decline, limiting the ability to draw causal conclusions. It is also important to note that our results do not account for the potential influence of other mental health disorders, which may further restrict the generalizability of our findings.

## 5 Conclusion

This study utilizes data from the CLASS survey conducted between 2016 and 2020 to systematically chart the diverse cognitive trajectories of older adults in China, focusing on the impact of multidimensional social isolation on these trajectories. Three distinct cognitive groups were identified among older adults: the High Baseline Stable Group, the High Baseline Declining Group, and the Low Baseline Deteriorating Group. The findings indicate that friend isolation and subjective isolation significantly increase the risk of both high-level cognitive decline and low-level deterioration, while the impact of family isolation appears minimal. Consequently, addressing friend and subjective isolation among older adults emerges as a critical strategy for proactively safeguarding cognitive health, which could substantially alleviate pressures on healthcare and social welfare systems. These insights are vital for developing targeted interventions aimed at enhancing cognitive well-being and contribute to broader efforts to mitigate the onset and progression of dementia and related conditions within the older adult demographic. Future research should incorporate more comprehensive assessment tools, ensure broader and more diverse sample representation, and extend the duration of follow-up.

## Data Availability

The datasets presented in this study can be found in online repositories. The names of the repository/repositories and accession number(s) can be found in the article/supplementary material.
